# *Echinococcus multilocularis* and Other Intestinal Parasites of the Red Fox (*Vulpes vulpes*) from the Pomerania Region, Northern Poland

**DOI:** 10.3390/pathogens13060490

**Published:** 2024-06-08

**Authors:** Bogumiła Pilarczyk, Agnieszka Tomza-Marciniak, Renata Pilarczyk, Małgorzata Bąkowska, Izabella Rząd, Agata Stapf, Lidia Felska-Błaszczyk, Agnieszka Tylkowska, Beata Seremak

**Affiliations:** 1 Department of Animal Reproduction Biotechnology and Environmental Hygiene, Faculty of Biotechnology and Animal Husbandry, West Pomeranian University of Technology, Janickiego 29, 71-270 Szczecin, Poland; bogumila.pilarczyk@zut.edu.pl (B.P.); agnieszka.tomza-marciniak@zut.edu.pl (A.T.-M.); beata.seremak@zut.edu.pl (B.S.); 2 Laboratory of Biostatistics, Faculty of Biotechnology and Animal Husbandry, West Pomeranian University of Technology, Janickiego 29, 71-270 Szczecin, Poland; renata.pilarczyk@zut.edu.pl; 3 Institute of Marine and Environmental Sciences, Molecular Biology and Biotechnology Centre, Faculty of Physical, Mathematical and Natural Sciences, University of Szczecin, Wąska 13, 71-415 Szczecin, Poland; izabella.rzad@usz.edu.pl; 4 Department of Biological Sciences, Faculty of Physical Culture in Gorzow Wielkopolski, Poznan University of Physical Education, Estkowskiego 13, 66-400 Gorzów Wielkopolski, Poland; a.stapfskiba@gmail.com; 5 Department of Animal Anatomy, Faculty of Biotechnology and Animal Husbandry, West Pomeranian University of Technology, Janickiego 33, 71-270 Szczecin, Poland; lidia.felska-blaszczyk@zut.edu.pl; 6 Department of Animal Environment Biology, Faculty of Animal Breeding, Bioengineering and Conservation, Warsaw University of Life Sciences, Ciszewskiego 8, 02-787 Warsaw, Poland; agnieszka_tylkowska@sggw.edu.pl

**Keywords:** *Alaria alata*, *Echinococcus multilocularis*, prevalence, red fox (*Vulpes vulpes*), *Toxocara canis*, zoonosis

## Abstract

The aim of the study was to determine the species composition of the intestinal parasite fauna of foxes from the Pomerania region, with a particular emphasis on helminth species considered dangerous to humans, and to determine their prevalence and intensity of infection. In total, 165 digestive systems from foxes inhabiting the Pomeranian region were examined. The prevalence of intestinal parasites among the studied foxes was 61.8%. Our findings confirm that foxes in Pomerania carry various parasites, some of which pose a direct threat to human health. As such, constant monitoring of their infestation is essential. Particular attention should be paid to parasite species with potential for transmission to humans, such as *Echinococcus multilocularis*, *Alaria alata* and *Toxocara canis*, whose respective prevalence was found to be 10.9%, 17.6% and 28.5%.

## 1. Introduction

The most common canine species in the Northern Hemisphere is the red fox (*Vulpes vulpes*). Although there has been an obvious decline in natural wildlife habitats in recent years as urbanisation has advanced, the fox is an extremely versatile predator that can adapt to a variety of environments, including urban areas. Their ability to utilise alternative food sources, such as anthropogenic food scraps, pet feed, carcasses of domestic animals and livestock, allows them to survive and reproduce even under harsh environmental conditions [1,2,3]. Studies have shown that foods of anthropogenic origin accounted for as much as 83.5% [1] and 74.8% [4] of the diet of foxes living in the suburbs of Zurich. These impressive results testify to the remarkable ability of the fox to adapt in changing environmental conditions.

Increasing number of foxes are migrating to urban environments, and their sightings are becoming increasingly common. This migration brings with it a new challenge for human health, as it increases the risk of contact between humans and foxes, and thus greater transmission of zoonotic parasites to both humans and their pets. One such challenge is posed by *Echinococcus multilocularis*, whose prevalence is increasingly being recorded in European cities. It has been detected in foxes in such locations as Copenhagen, Zurich, Geneva and Stuttgart [5,6,7].

When food is plentiful, a fox may roam over several hectares. However, this territory can extend to more than 2000 or 3000 hectares (ha) when food is scarce [8].

Over the past 30 years, the fox population in Poland has more than tripled. Estimates suggest that it numbered around 56,000 in 1990, and this had risen to an impressive 188,000 in 2022 [9]. It is possible that this significant increase may be a direct result of the fox anti-rabies vaccination programme that has been in place since 1993. Currently, vaccinations are performed in forest areas and any urban locations inhabited by free-living foxes. They are typically administered twice a year, typically in spring and autumn; however, if no sign of rabies has been detected in the voivodeship for two consecutive years, they may be administered once a year. If no sign of rabies has been noted for three consecutive years, no vaccination is performed [10]. This large-scale programme has contributed to a significant reduction in rabies mortality among foxes, which would translate into an increase in their numbers.

This increase in the fox population in Poland carries with it a number of risks, including an elevated risk of zoonotic transmission of parasitic agents such as *Toxocara canis* and *Echinococcus multilocularis*. As such, there is a need to update the research on the intestinal parasite fauna of these predators.

We therefore hypothesise that this increase in the fox population in Poland, resulting from the successful rabies vaccination programmes, led to an increase in the number of parasites in foxes and thus an increased risk of transmission of parasitic diseases caused by *Toxocara canis* or *Echinococcus multilocularis* to humans.

The aim of the study was therefore to determine the species composition of the parasitofauna of foxes from the Pomerania region, with a particular emphasis on helminth species considered dangerous to humans. The study also determined the prevalence and intensity of infection with the identified parasites.

## 2. Materials and Methods

### 2.1. Natural Characteristics of the Sampling Regions

#### 2.1.1. Western Pomerania

**Figure 1:**
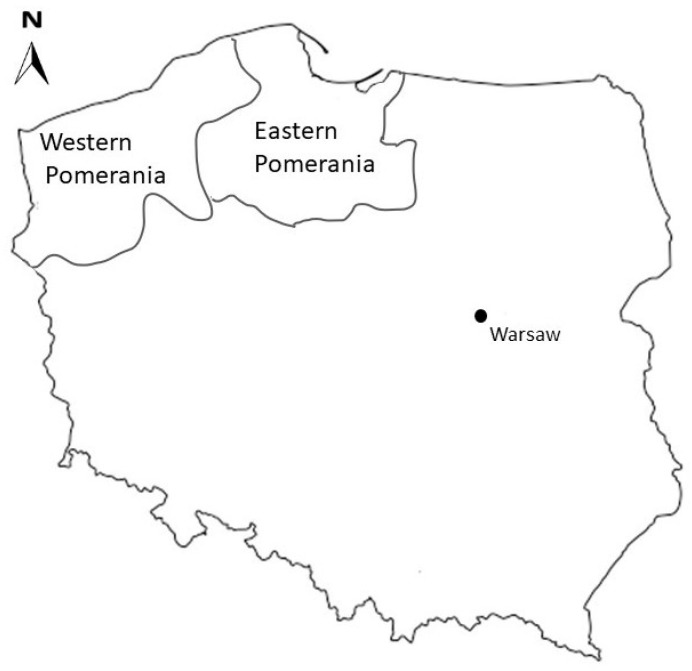
Location of the study area.

Western Pomerania comprises the borders of the former Duchy of Pomerania and the areas of the former province of Pomerania, and now includes the province of West Pomerania. The region presents the characteristic relief of a post-glacial area, and has one of the richest levels of plant biodiversity in Poland. It encompasses aquatic, peatland, meadow and even steppe ecosystems as well as forest ecosystems. The degree of forest cover in the region is significant (35.7%). However, the distribution of forests ranges from less than 1% to more than 70% of the areas of the province, with those in the south-eastern part of the region having the highest forest cover. In unmanaged forests, the forest stand is mainly represented by deciduous trees (i.e., beech, oak) and mixed forests with admixture of pine, oak-hornbeam forests, riparian forests and alder forests. Protected plants such as the royal longhorn and crow’s-foot trefoil can also be found. The specific post-glacial relief of the area is associated with a wide variety of biotopes providing habitats for animals. The West Pomeranian region includes a long Baltic coastline that encompasses the islands of Usedom and Wolin, a number of lakes and rivers and the marshy area of the lower course of the Oder River, making it an excellent habitat for diverse fauna (Figure 1).

#### 2.1.2. Eastern Pomerania

Eastern Pomerania, also known as the Gdansk Pomerania, stretches from the Slupia basin to the Vistula delta. Administratively, it includes the entire Pomeranian Voivodeship and the area adjacent to the Vistula Lagoon, as well as part of the Warmian-Masurian Voivodeship. The relief, like that of Western Pomerania, is a post-glacial area. Around 32% of the land is covered by forest, and another 32% by agricultural land. The territory of eastern Pomerania is characterised by a considerable diversity of environmental types, often with characteristics of natural habitats. Its adjacency to the waters of the Baltic Sea results in the presence of strip dunes, cliff shores, river valleys and extensive mudflats. Eastern Pomerania also includes the Vistula delta plain with its rich vegetation and habitat which serves a range of animal species. The area encompasses numerous lakes and spring areas, giving rise to many of the watercourses in the region. It is also home to a number of peat bogs, which act as retainers, providing a refuge for many declining and endangered plant, fungal and animal communities (Figure 1).

### 2.2. Animals

The study was performed on the digestive systems of 165 foxes taken by hunters during hunts between September 2018 and October 2022. These animals inhabited Pomerania, with 68 coming from Western Pomerania (WP) and 97 from Eastern Pomerania (EP). All foxes were weighed and measured. Their age was determined by the degree of tooth wear [8]. Animals less than one year old were classified as juveniles, and those over one year old as adults.

The foxes were obtained during hunting programmes aimed at reducing the fox population in the Pomerania area. According to ACT of 15 January 2015 on the protection of animals used for scientific or educational purposes [11], the collection of tissues from animals killed for other than scientific and didactic reasons is not classified as experimental work, and therefore does not require ethics committee approval.

### 2.3. Parasitological Analysis

The intestines were analysed using the sedimentation and counting technique (SCT) [12]. Each intestine was cut lengthwise and checked for the presence of adult parasites; it was then divided into 20 cm long sections, and each section was rinsed in physiological saline solution (1 L). The mucosa was then manually removed, and the intestine removed from the flask in which the rinsing took place. The obtained suspension of intestinal material in physiological saline was left to sediment multiple times for 15 min. Each time, the supernatant was carefully poured off and sediment was transferred in small increments onto Petri dishes and examined using a stereomicroscope.

Parasites were identified based on morphological features such as dimensions, shape and structural features, using the keys described by Khalil et al. [13], Yamaguti [14], Bray et al. [15] and Anderson et al. [16].

### 2.4. Statistical Analysis

The results were analysed with using Statistica 13.0 (TIBCO Software Inc., Palo Alto, CA, USA). Differences between young and adult foxes and between males and females in the prevalence of individual parasites, and the intensity of infection were assessed using the χ^2^ test and the Mann–Whitney U-test, respectively. Differences were determined to be statistically significant at *p* < 0.05. The confidence interval of a proportion was calculated using the modified Wald method [17].

## 3. Results

The prevalence of parasite infection among foxes in Pomerania was 61.8% (Table 1). A much higher prevalence (67%) was recorded in foxes from East Pomerania (Table 2). The obtained intestinal parasite fauna included: *Echinococcus multilocularis* (10.9%), *Toxocara canis* (28.5%), *Toxascaris leonina* (11.5%), *Alaria alata* (17.6%), *Teania* spp. (15.8%), *Uncinaria stenocephala* (23.6%), *Mesocestoides* spp. (40.0%) as well as *Dipylidium caninum* (5.5%). The most common parasite found in foxes from Pomerania was *Mesocestoides* spp. (40%), and the least frequent was *Dipylidium caninum* (5.5%) (Table 1).

Foxes from East Pomerania had a significantly (*p* < 0.01) higher prevalence of *Echinococcus multilocularis* (χ^2^ = 7.6; *p* = 0.006) than those from West Pomerania. However, no significant differences in the intensity of infection with individual parasite species were noted between the two areas (Table 2).

Young foxes demonstrated a significantly higher prevalence of *Toxocara canis* (χ^2^ = 9.6; *p* = 0.002), while adult foxes were significantly more likely to present *Alaria alata* (χ^2^ = 4.2; *p* = 0.04) and *Mesocestoides* spp. (χ^2^ = 9.4; *p* = 0.002) infection (Table 3).

Adult foxes had a significantly higher mean intensity of infection with *Echinococcus multilocularis* (U = 7.0; *p* = 0.01) and *Alaria alata* (Z = −2.42; *p* = 0.01). They also demonstrated a higher overall mean intensity of parasite infection (Z = −2.06; *p* = 0.04) (Table 3). No significant differences in the prevalence or intensity of parasite infection were found according to host sex (Table 4). The majority of foxes had single-species and two-species infections (Table 5).

## 4. Discussion

Among the known parasitic zoonoses, is one of the most serious is human alveolar echinococcosis, caused by the larvae of the tapeworm *Echinococcus multilocularis* from the Taeniidae family. In such cases, humans serve as an accidental intermediate host in the *E. multilocularis* developmental cycle. Due to its serious risk to human health and life, echinococcosis has been identified as a public health priority, and in the European Union, it is one of eight zoonoses subject to mandatory monitoring. The red fox is widely recognised as the main species responsible for environmental contamination in Europe with invasive *E. multilocularis* eggs [18]. As such, it would be beneficial to determine its prevalence in the fox population, as this would provide an indirect assessment of the risk of infection for humans.

In the present study, the prevalence of *E. multilocularis* infestation in foxes in Pomerania was 10.91%. This result is lower than that noted by Karamon et al. [19], who reported a prevalence of 16.5% in Poland.

Our findings indicate that foxes from East Pomerania were more frequently infected with *E. multilocularis* than those from West Pomerania, with the prevalence being 2.9% in Western Pomerania (two individuals) and 16.5% in Eastern Pomerania (16 individuals). These observations are in line with the uneven distribution of *E. multilocularis* infestations across Poland as a whole [19]. The authors identified a clear correlation between the occurrence of *E. multilocularis* in foxes and the geographical region in Poland. The highest infection rates were recorded in the Warmińsko-Mazurskie (*Warmia-Masuria*) and Podkarpacki (*Subcarpathian*) provinces, where the parasite was found in around 50% of the studied foxes.

The situation in the western part of Poland was radically different, with a much lower prevalence that did not exceed a few percent. Karamon et al. [19] indicate that although a fourfold nationwide increase in the fox population has been recorded in Poland over the past several years, the proportion of foxes infected with *E. multilocularis* has only increased in the eastern part of Poland; in the west, the level of invasion remains relatively low. One of the factors contributing to the higher prevalence in Eastern Pomerania may be the high prevalence of *E. multilocularis* in foxes in the Warmia-Masuria voivodeship (50%).

Foxes show impressive mobility, potentially covering straight-line distances in excess of 108 km in just seven days [20]. Such migration can encourage the transmission of parasites from areas of higher prevalence to areas of lower prevalence. Recent decades have also seen an increase in the prevalence of *E. multilocularis* in European foxes, with particularly clear upward trends being observed in Germany: over a 15–20 year period, the proportion of infected foxes increased from 12% to 40% in central Germany [21] and from 12% to 20% in northern Germany [22].

The density of the fox population in Poland is estimated to be 7.8 individuals per 1000 ha [23]. This is much higher than the target density, which is widely believed to be between one and three animals per 1000 ha [8]. At such high population densities, the risk of *E. multilocularis* transmission can be significant. In fact, as the overall prevalence exceeds 10% of all foxes, Poland is regarded as a high-endemic country [18] together with the Czech Republic, Estonia, France, Germany, Latvia, Lithuania, Slovakia, Liechtenstein and Switzerland.

The literature suggests that *Alaria alata* may also pose a risk to humans, who may become intermediate hosts after consuming food of animal origin containing the mesocercariae. Such high-risk foods include undercooked frogs’ legs and raw or semi-raw wild boar meat products [24,25]. The prevalence of *A. alata* in red foxes varies widely between countries in Europe, i.e., from 1.2% in Slovakia [26] to 5.3% in Italy [27], 25.6% in Serbia [28], to as high as 94.8% in Lithuania [29].

Our present findings indicate a prevalence of 17.6% in the studied foxes. This result is lower than the infection rate observed by other researchers in Poland. Tylkowska et al. [30] report an infestation rate of 54.7% in surveyed foxes from north-western Poland. Also, Karamon et al. [31] found the prevalence ranging from 15.2% in the south to 90% in the north of the country. The authors attribute this relationship to the high availability of surface water in the north, which is essential for the parasite to thrive: high levels of *A. alata* infection are noted among local wildlife in humid areas inhabited by snails and amphibians, i.e., its definitive and paratenic hosts. Further regional variation was noted by Karamon et al. [32], who report a significantly higher infection rate (78.7%) in foxes from eastern Poland (Lublin Voivodship).

Nematodes of the genus *Toxocara* are non-animal soil-transmitted parasites with high socioeconomic importance [33]. *Toxocara* is an example of a parasite that can be transmitted between wild canids, domesticated animals such as cats and dogs, and humans. In most cases, human toxocariasis and its associated complications are due to *Toxocara canis* and to a lesser extent, *Toxocara cati*. The clinical forms of toxocariasis demonstrate considerable diversity. The generalized form includes visceral larva migrans syndrome (VLM), ocular larva migrans (OLM) and neurological larva migrans (NLM), as well as covert toxocariasis [34].

Our data indicate the prevalence of *T. canis* in foxes to be 28.5%. This high presence may suggest that these animals are significantly contributing to the contamination of the environment with *Toxocara* eggs. As such, foxes that approach human dwellings pose a particular threat to people and their pets by potentially exposing them to the parasite through their faeces.

Studies have found *T. canis* infection rates in foxes to vary significantly between across Europe. While some countries have demonstrated similar rates to our present findings, such as Croatia with 28.2% [35], Germany with 31.3% [36] and Belarus with 25.5% [37], lower infection rates were reported in Italy with 9.1% [38], and others have demonstrated much higher rates of infection: the UK, i.e., 61.6% [39], the Netherlands, 61% [40], Denmark, around 60.9% [41], Switzerland, 44.3% [42] and Slovenia, 38.3% [43].

In Poland, between 1994 and 2005, between 16.6% and 75.6% of seropositive human cases were reported among individuals suspected of *Toxocara* infection [44,45]. The prevalence of anti-*T. canis* serum antibody varies between different regions. The prevalence identified in Poland is comparable to that observed in other European countries, e.g., 32.6% in Bulgaria and 25.8% in Austria [46,47]. However, these results differ significantly from those recorded in Slovakia (3.7%) [48], in Japan (1–6%), Denmark (6%), Austria (7%) and Nepal (81%) [34]. In turn, studies performed in the period 2009–2011 by Mazur-Melewska et al. [49] recorded a 38% prevalence of seropositivity among children in Poland; these results are comparable to those obtained in children in Croatia (31%) [50].

In the present study, no significant differences in nematode prevalence were observed between male and female foxes. These results are in line with those of previous studies, which also found no relationship between sex and parasite prevalence [51,52,53,54]. This lack of differences may be due to the fact that male and female foxes share a similar diet. In addition, male and female foxes have similar defence mechanisms against intestinal parasites, live in similar habitats and have similar degrees of contact with potential sources of infection.

In our study, younger foxes showed significantly higher levels of *Toxocara canis* infection then older animals. This may be due to the fact that in canids, the transmission of reactivated larvae occurs through the placenta and, to a lesser extent, through the lactogenic route [55]. In addition, Reperant et. al. [42] report a higher prevalence of *Toxocara canis* infection among foxes under six months of age.

## 5. Conclusions

Significant changes in the intestinal parasite fauna of foxes living in Pomerania have been observed in recent years, with both the prevalence of individual parasite species increasing, as well as the species composition itself. Additionally, the prevalence of individual parasites differs between foxes in East and West Pomerania, which may reflect variation in environmental factors.

Foxes in Pomerania carry many parasites, some of which pose a direct threat to human health. Of particular concern is the high prevalence of *Echinococcus multilocularis* in foxes in Eastern Pomerania, which may be linked to local environmental factors.

With the increasing zoonotic threat posed by the parasites carried by foxes, constant monitoring of their presence and infection level is essential. Such studies should focus on the parasite species with potential for transmission to humans, such as *Echinococcus multilocularis*, *Alaria alata* and *Toxocara canis*.

## Figures and Tables

**Table 1:** Prevalence of intestinal parasites in red foxes (N = 165) in Pomerania, Poland.

	n	%	95% CI
*Echinococcus multilocularis*	18	10.9	6.6–16.7
*Toxocara canis*	47	28.5	22.1–35.8
*Toxascaris leonina*	19	11.5	7.4–17.4
*Alaria alata*	29	17.6	12.5–24.2
*Taenia* spp.	26	15.8	10.9–22.1
*Uncinaria stenocephala*	39	23.6	17.8–30.7
*Mesocestoides* spp.	66	40.0	32.8–47.6
*Dipylidium caninum*	9	5.5	2.8–10.2
Total	102	61.8	54.2–68.9

n—number of positive animals; N—number of tested animals.

**Table 2:** Prevalence of intestinal parasites in red foxes in Western and Eastern Pomerania, Poland.

Parasite	Area	Number of Foxes Infected/Tested	Prevalence (%) (95% CI)	χ^2^ Test Value	Intensity of Infection				
					Mean	GM	Median	Range	Mann–Whitney U-Test Value
*Echinococcus multilocularis*	WP	2/68	2.9 (0.2–10.7)	χ^2^ = 7.6; *p* = 0.006	11	11	11	8–14	U = 9.0 Z = −0.91 *p* = 0.39
	EP	16/97	16.5 (10.3–25.2)		21	16	17	5–50	
*Toxocara canis*	WP	20/68	29.4 (19.9–41.2)	χ^2^ = 0.05; *p* = 0.82	8	8	8	2–14	U = 207.5 Z = −1.35 *p* = 0.18
	EP	27/97	27.8 (19.9–37.5)		10	10	9	6–20	
*Toxascaris leonina*	WP	5/68	7.4 (2.8–16.5)	χ^2^ = 2.0; *p* = 0.16	8	7	8	3–12	U = 27.0 Z = −0.69 *p* = 0.50
	EP	14/97	14.4 (8.7–22.9)		10	9	10	2–16	
*Alaria alata*	WP	9/68	13.2 (6.9–23.5)	χ^2^ = 1.5; *p* = 0.22	22	17	19	6–45	U = 72.5 Z = 0.80 *p* = 0.42
	EP	20/97	20.6 (13.7–29.8)		18	14	13	5–70	
*Taenia* spp.	WP	11/68	16.2 (9.1–26.9)	χ^2^ = 0.02 *p* = 0.9	3	2	2	1–6	U = 55.00 Z = −1.44 *p* = 0.15
	EP	15/97	15.5 (9.5–24.1)		5	4	4	1–15	
*Uncinaria stenocephal*	WP	13/68	19.1 (11.4–30.2)	χ^2^ = 1.3; *p* = 0.25	8	5	6	1–20	U = 368.5 Z = 0.02 *p* = 0.99
	EP	26/97	26.8 (19.0–36.4)		7	6	6	1–18	
*Mesocestoides* spp.	WP	22/68	32.4 (22.4–44.2)	χ^2^ = 2.8; *p* = 0.09	16	11	16	1–51	U = 368.50 Z = 1.58 *p* = 0.11
	EP	44/97	45.4 (35.8–55.3)		12	6	9	1–42	
*Dipylidium caninum*	WP	5/68	7.4 (2.8–16.5)	χ^2^ = 0.8; *p* = 0.37	3	3	4	2–4	U = 6.5 Z = −0.73 *p* = 0.41
	EP	4/97	4.1 (1.3–10.5)		5	4	5	1–10	
Total	WP	37/68	54.4 (42.7–65.7)	χ^2^ = 2.7; *p* = 0.10	25	19	17	3–72	U = 1063.0 Z = −0.97 *p* = 0.33
	EP	65/97	67.0 (57.1–75.6)		30	20	25	1–146	

WP—Western Pomerania; EP—Eastern Pomerania; GM—Geometric mean.

**Table 3:** Prevalence of intestinal parasites in red foxes in Pomerania, Poland, in relation to host age.

Parasite	Age	Number of Foxes Infected/Tested	Prevalence (%) (95% CI)	χ^2^ Test Value	Intensity of Infection				
					Mean	GM	Median	Range	Mann–Whitney U-Test Value
*Echinococcus multilocularis*	Young	5/74	6.8 (2.6–15.2)	χ^2^ = 2.4; *p* = 0.12	8	8	9	5–11	U = 7.0 Z = −2.46 *p* = 0.01
	Adult	13/91	14.3 (8.4–23.1)		24	20	17	8–50	
*Toxocara canis*	Young	30/74	40.5 (30.1–51.9)	χ^2^ = 9.6; *p* = 0.002	10	9	9	2–20	U = 230.5 Z = 0.54 *p* = 0.59
	Adult	17/91	18.7 (11.9–28.0)		9	9	9	6–12	
*Toxascaris leonina*	Young	10/74	13.5 (7.3–23.3)	χ^2^ = 0.5; *p* = 0.47	8	7	10	2–14	U = 30.0 Z = −1.18 *p* = 0.24
	Adult	9/91	9.9 (5.1–17.9)		11	10	12	6–16	
*Alaria alata*	Young	8/74	10.8 (5.4–20.2)	χ^2^ = 4.2; *p* = 0.04	10	9	10	5–19	U = 34.0 Z = −2.42 *p* = 0.01
	Adult	21/91	23.1 (15.6–32.8)		23	18	15	6–70	
*Taenia* spp.	Young	10/74	13.5 (7.3–23.3)	χ^2^ = 0.5; *p* = 0.48	5	4	4	1–15	U = 70.0 Z = 0.51 *p* = 0.61
	Adult	16/91	17.6 (11.0–26.8)		4	3	2	1–9	
*Uncinaria stenocephal*	Young	14/74	18.9 (11.5–29.4)	χ^2^ = 1.7; *p* = 0.20	8	6	6	1–20	U = 173.0 Z = 0.04 *p* = 0.96
	Adult	25/91	27.5 (19.3–37.5)		7	6	4	1–20	
*Mesocestoides* spp.	Young	20/74	27.0 (18.2–38.2)	χ^2^ = 9.4; *p* = 0.002	16	11	15	1–51	U = 363.0 Z = 1.36 *p* = 0.17
	Adult	46/91	50.6 (40.5–60.6)		12	6	12	1–42	
*Dipylidium caninum*	Young	3/74	4.0 (0.9–11.7)	χ^2^ = 0.5; *p* = 0.48	6	5	4	4–10	U = 4.0 Z = 1.16 *p* = 0.26
	Adult	6/91	6.6 (2.8–13.9)		3	3	3	1–5	
Total	Young	45/74	60.8 (49.4–71.2)	χ^2^ = 0.06; *p* = 0.81	22	18	20	3–72	U = 976.0 Z = −2.06 *p* = 0.04
	Adult	57/91	62.6 (52.4–71.9)		33	22	28	1–146	

GM—Geometric mean.

**Table 4:** Prevalence of intestinal parasites in red foxes in Pomerania, Poland, in relation to host sex.

Parasite	Sex	Number of Foxes Infected/Tested	Prevalence (%) (95% CI)	χ^2^ Test Value	Intensity of Infection				
					Mean	GM	Median	Range	Mann–Whitney U-Test Value
*Echinococcus multilocularis*	♂	9/82	11.0 (5.7–19.8)	χ^2^ = 0.0; *p* = 0.98	19	17	16	9–45	U = 34.5 Z = 0.49 *p* = 0.60
	♀	9/83	10.8 (5.6–19.6)		20	15	10	5–50	
*Toxocara canis*	♂	23/82	28.1 (19.4–38.6)	χ^2^ = 0.02; *p* = 0.90	9	8	8	2–20	U = 226.5 Z = −1.05 *p* = 0.30
	♀	24/83	28.9 (20.2–39.5)		10	9	9	3–20	
*Toxascaris leonina*	♂	8/82	9.8 (4.8–18.3)	χ^2^ = 0.5; *p* = 0.48	9	8	9	3–14	U = 40.5 Z = −0.25 *p* = 0.78
	♀	11/83	13.3 (7.4–22.4)		10	9	10	2–16	
*Alaria alata*	♂	15/82	18.3 (11.3–28.1)	χ^2^ = 0.06; *p* = 0.81	18	15	14	5–45	U = 100.0 Z = 0.20 *p* = 0.84
	♀	14/83	16.9 (10.2–26.5)		21	15	13	6–70	
*Taenia* spp.	♂	12/82	14.6 (8.4–24.0)	χ^2^ = 0.2; *p* = 0.69	4	3	2	1–8	U = 76.0 Z = −0.40 *p* = 0.69
	♀	14/83	16.9 (10.2–26.5)		5	3	4	1–15	
*Uncinaria stenocephal*	♂	23/82	28.1 (19.4–38.6)	χ^2^ = 1.6; *p* = 0.18	7	6	6	2–20	U = 183.5 Z = 0.00 *p* = 1.00
	♀	16/83	19.3 12.1–29.2)		8	5	6	1–20	
*Mesocestoides* spp.	♂	36/82	43.9 (33.7–54.7)	χ^2^ = 1.0; *p* = 0.31	13	7	13	1–51	U = 512.0 Z = −0.32 *p* = 0.72
	♀	30/83	36.1 (26.6–46.9)		14	8	11	1–42	
*Dipylidium caninum*	♂	6/82	7.3 (3.1–15.4)	χ^2^ = 1.1; *p* = 0.30	4	3	4	1–10	U = 7.5 Z = 0.26 *p* = 0.71
	♀	3/83	3.6 (0.8–10.5)		3	3	4	2–4	
Total	♂	56/82	68.3 (57.6–77.4)	χ^2^ = 2.9; *p* = 0.09	25	18	23	1–72	U = 1151.5 Z = −0.91 *p* = 0.36
	♀	46/83	55.4 (44.7–65.6)		31	22	25	1–146	

GM—Geometric mean.

**Table 5:** Occurrence of mixed infection of intestinal parasites in red foxes (N = 165) in Pomerania, Poland.

Number of Parasite Species	WP		EP		Total	
	n	%	n	%	n	%
0	31	45.6	32	33.0	63	38.2
1	12	17.6	16	16.5	28	17.0
2	11	16.2	20	20.6	31	18.8
3	3	4.4	12	12.4	15	9.1
4	8	11.8	11	11.3	19	11.5
5	1	1.5	6	6.2	7	4.2
6	2	2.9	0	0.0	2	1.2
Total	68		97		165	

WP—Western Pomerania; EP—Eastern Pomerania; n—number of tested animals.

## Data Availability

The raw data supporting the conclusions of this article will be made available by the authors on request.
